# Patient Experiences of Chronic Low Back Pain: Development of a Conceptual Model Based on a Targeted Literature Review and Qualitative Patient Interviews

**DOI:** 10.1002/msc.70281

**Published:** 2026-10-28

**Authors:** Ekin Seçinti, Brittany Klooster, Rebecca Robinson, Madison C. Bernstein, Madeline Tallarico, Kaitlin Chatterton, Adriana Voci, Daniella Olonilua, Samantha R. Linton, Leighann Litcher‐Kelly, Marcia M. Mellado Lagarde, Linda A. Wietecha, John Markman

**Affiliations:** ^1^ Eli Lilly and Company Indianapolis Indiana USA; ^2^ Adelphi Values Boston Massachusetts USA; ^3^ Formerly Adelphi Values Boston Massachusetts USA

**Keywords:** chronic low back pain, CLBP, clinical outcome assessment, literature review, patient experience, qualitative interviews

## Abstract

**Purpose:**

Chronic low back pain (CLBP) is a persistent, debilitating condition. The United States (US) Food and Drug Administration Patient‐Focused Drug Development Guidance recommends using patient‐reported outcome (PRO) measures to assess pain and function in clinical trials and document content validity of the measures based on patient experience data. The objective of this study was to explore and document the patient experience of symptoms and health‐related quality of life (HRQoL) impacts of CLBP based on a targeted literature review (TLR) and qualitative patient interviews to facilitate future PRO validation studies.

**Methods:**

A TLR of patient‐focused studies and qualitative interviews (*N* = 30) with adults with CLBP in the US were conducted to document the patient perspective of CLBP symptoms and HRQoL impacts. Results were combined in a patient‐centred conceptual model.

**Results:**

Merged results from the TLR and qualitative interviews demonstrated that adults with CLBP described highly impactful experiences of pain. The conceptual model grouped 16 symptom and 120 impact concepts. CLBP was described as commonly co‐occurring with numbness, muscle spasms, and stiffness. CLBP‐related HRQoL impacts included physical functioning, emotional functioning, adaptive behaviours, sleep, social functioning, household chores/responsibilities, and work/school.

**Conclusion:**

The findings highlight the multidimensional burden of living with CLBP. The newly developed patient‐centred conceptual model may be used to explore the content validity of PRO measures and promote a robust understanding of the patient experience with CLBP. Findings suggest that utilising HRQoL endpoints and patient‐centred approaches in CLBP studies of novel therapies may be an important way to quantify meaningfully effective treatments.

## 1 Introduction

Chronic low back pain (CLBP) is defined as musculoskeletal pain lasting longer than three months between the 12th thoracic vertebra and the lower gluteal folds (Nicol et al. 2023). As of 2020, CLBP is the most prevalent musculoskeletal condition globally affecting 619 million people worldwide (Ferreira et al. 2023), and the most prevalent pain syndrome in the United States (US). This condition is a leading cause of years of disability (Mokdad et al. 2018; Nieminen et al. 2021). Patients with CLBP commonly experience pain attacks (i.e., fluctuations in pain intensity), as well as pressure, muscle tension, prickling, numbness, and stiffness. Pain is often characterised as muscle aches or a shooting, burning, or stabbing sensation that can radiate down the legs (Hider et al. 2015; Markman et al. 2020; Martin et al. 2018; Morlion 2013). These symptoms have wide‐ranging effects on behaviour, functioning, and health‐related quality of life (HRQoL) and are associated with decreased work productivity, poor mental health (e.g., anxiety, depression), and higher healthcare costs (Fullen et al. 2022; Hartvigsen et al. 2018; Hider et al. 2015; Hush et al. 2009; Urits et al. 2019).

Current clinical goals of CLBP treatment are focused on pain management, as the available pharmacological treatments (e.g., muscle relaxants, analgesics, steroid injections) often do not successfully resolve the pain associated with CLBP (Chou, Deyo, Friedly, Skelly, Hashimoto, et al. 2017; Chou, Deyo, Friedly, Skelly, Weimer, et al. 2017; Morlion 2013; Patel et al. 2015; Qaseem et al. 2017). Hence, there is an unmet need for more comprehensive treatments that aim to reduce the pain and functional limitations associated with CLBP (Knezevic et al. 2021). New treatment options are needed to reduce disability in addition to alleviating pain, and using a multi‐dimensional treatment approach could improve patients' overall quality of life (Knezevic et al. 2021; National Academies of Sciences et al. 2017).

As an essential part of patient‐focused drug development, the Food and Drug Administration (FDA) Patient‐Reported Outcomes (PRO) and Patient‐Focused Drug Development (PFDD) Guidance documents recommend clinical trials to evaluate treatment benefit using clinical outcome assessments (COAs) measuring how patients feel, function, or survive (US Department of Health and Human Services et al. 2025). Pain is a multidimensional and subjective experience, and research to understand the patient experience of CLBP is a fundamental step in identifying the relevant concepts of interest (i.e., symptoms and HRQoL impacts) to support the development or selection of PRO measures to be used in clinical trials for CLBP treatments (US Department of Health and Human Services et al. 2025). PRO measures for use in clinical trials should assess what is important and relevant to patients. The National Institutes of Health (NIH) Pain Consortium's Research Task Force established a foundational methodological framework for CLBP research, providing consensus recommendations on core outcome domains, including patient‐reported pain intensity, pain interference, physical function, depression, sleep disturbance, and catastrophizing (Deyo et al. 2015). Building on this framework, the present study uses qualitative methods to generate current patient experience evidence to inform the content validity of PRO measures aligned with established research standards.

Qualitative evidence of patient experience, as summarised in a conceptual model, can be used to evaluate the content validity of a PRO measure, documenting whether the PRO measure is conceptually relevant and comprehensive for this population (US Department of Health and Human Services et al. 2025). While research into the patient experience of CLBP has been published, it is important to ensure that detailed, updated insights into the concepts most important to patients with CLBP have been obtained using qualitative research methods. Several reviews focussing on the patient experience of CLBP have been published (Toye et al. 2013). However, these findings are over 10 years old, and these reviews did not include patient‐centred conceptual models of the symptoms and HRQoL impacts of CLBP in line with current regulatory standards.

The overall objective of this study was to explore and document the patient perspective of the key symptoms and the associated HRQoL impacts of CLBP through a targeted review of the published literature and conduct of qualitative interviews with patients. These activities aimed to develop a patient‐centred conceptual model of CLBP, which can be used to explore the content validity of PRO measures and enhance understanding of the patient experience of living with CLBP.

## 2 Methods

### 2.1 Study Methodology Overview

This study involved a targeted literature review (TLR) and subsequent qualitative interviews with adults (aged ≥ 18 years) with CLBP. Based on the TLR findings, an initial draft of the conceptual model was developed. Upon completion of the qualitative interviews, the TLR concepts and those elicited during the qualitative interviews were combined to create a final patient‐centred conceptual model reflecting the complete findings from the TLR and qualitative interviews, which aligned with the FDA's PFDD Guidance (US Department of Health and Human Services et al. 2025).

### 2.2 Targeted Literature Review

A protocol for the TLR was developed prior to initiating any research activities. This protocol specified the procedures for creating and implementing the targeted search strategy, selecting articles, and extracting relevant data from those articles.

A targeted search was conducted on the MEDLINE, EMBASE, and APA PsycINFO databases via the OvidSP platform. The search strategy included terms to identify relevant qualitative research articles published in the last 10 years (i.e., published between December 2013 and January 2023) to focus on the contemporary literature reporting the symptoms and HRQoL impacts associated with CLBP from a patient perspective. Details of the search strategy are provided in Supporting Information S1: Table S1.

Abstracts were screened using Abstrackr (Agency for Healthcare Research and Quality 2017), a web‐based programme that allows researchers to review abstracts using eligibility criteria (Supporting Information S1: Table S2). Each abstract was screened by one member of the research team, and then all research team members reviewed screening decisions together until agreement was reached. The reference lists of abstracts selected for full review from the OvidSP search were reviewed to identify additional relevant articles or resources. Key criteria for reviewing articles from all sources included: peer‐reviewed research conducted in adult populations (≥ 18 years old), data pertaining to the symptoms and/or HRQoL impacts of CLBP, and data collected using qualitative methods.

Data extracted from selected full‐text articles included information about the study design and salient information on the CLBP‐related symptoms and HRQoL impact concepts reported by patients. Concept description tables were developed to summarise the concepts identified in the literature.

### 2.3 Qualitative Patient Interviews

An interview sample size of 30 participants was selected a priori to achieve saturation across concepts reported in the sample (i.e., the point at which no new concept elicitation‐based information would be gained from conducting additional interviews) in line with best practice recommendations (Lamoureux et al. 2015; Turner‐Bowker et al. 2018; US Department of Health and Human Services et al. 2020).

Observational cross‐sectional qualitative interviews were conducted with adults with a clinician‐confirmed diagnosis of CLBP living in the US. The qualitative interview eligibility (inclusion/exclusion) criteria are described in Supporting Information S1: Table S3. Participants were recruited by a professional healthcare research recruitment agency. Recruitment targets were created to ensure diversity of the sample in terms of sex, race, ethnicity, and education level (US Department of Health and Human Services et al. 2020). Interview participants self‐reported demographic and health information.

Participants completed online qualitative interviews, lasting up to 90 minutes, between 23 August and 11 November 2023. Participants were assigned unique identifiers to protect their privacy. A semi‐structured interview guide developed for this study was used to elicit descriptions of their experience with CLBP, including the symptoms and HRQoL impacts. Open‐ended questions were used to elicit spontaneous discussion of CLBP experiences (e.g., *‘Can you tell me about the symptoms you experience or have experienced due to CLBP?’; ‘How does pain impact your daily life?’*). Participants were prompted about specific HRQoL impact concepts identified in the TLR if they did not discuss these unprompted (e.g., *‘Has back pain impacted your ability to work/go to school?’; ‘Has CLBP impacted your ability to lift or carry objects?’*). These prompts were specific to elicit information about the HRQoL impacts of CLBP. Participants received an honorarium for their participation in the study. Interviews were audio‐recorded, transcribed, and de‐identified prior to coding and analysis.

Demographic and health information were summarised descriptively. Experienced COA researchers conducted qualitative analyses of de‐identified transcripts using ATLAS.ti software (Friese 2019; Lewins and Silver 2007). Qualitative data analysis involves coding, which is an iterative process by which researchers assign descriptive labels to interview text (single words, phrases, or longer stretches of speech) based on the study's research questions or goals (Patrick et al. 2011). Prior to coding, a codebook, based on the semi‐structured interview guide, containing a list of all the codes used to characterise important segments of transcript text was developed to guide and organise the content and meaning of actual participant language during analysis. Seven COA researchers coded the interview transcripts and subsequently met with the senior members of the research team to review any specific questions identified by the researchers and the final code list, which was updated during the iterative coding process. The full research team aligned on the final list of codes and resolved any outstanding questions. Concept saturation was assessed to confirm that an adequate sample size had been recruited (i.e., no new concept elicitation‐based information would be gained from conducting additional interviews) (Turner‐Bowker et al. 2018).

## 3 Ethics Approval

All study documents were submitted to a centralised independent review board (IRB) at Sterling IRB, and received approval on 10 August 2023 (IRB ID#: 11176‐KChatterton). All participants provided written informed consent before participating.

### 3.1 Merging Patient‐Experience Data and Development of the Conceptual Model

Concepts identified from the TLR and qualitative patient interviews first were qualitatively analysed and described independently based on the respective source material. After which, the results from the TLR and the qualitative patient interviews were compared, and homogeneous concepts were merged based on similar patient‐reported and literature‐based descriptions. All results were combined into a comprehensive conceptual model, aligned with recommended methods (Patrick et al. 2011) to characterise the CLBP patient experience (i.e., symptoms and HRQoL impacts) and guide measurement strategy. The conceptual model presented the overlapping symptom and HRQoL impact concepts across these two research activities, in alignment with the FDA's PFDD Guidance (US Department of Health and Human Services et al. 2025).

## 4 Results

### 4.1 Targeted Literature Review

In total, 295 records were identified from the database search (Figure 1); 284 records were excluded during title and abstract screening and three records were excluded during full‐text screening. Nine additional records were identified via hand‐searching of reference lists and were included in the final list of eligible studies. In total, 17 publications were selected for inclusion in the review (Bailly et al. 2015; Bushnell et al. 2018; Calmon Almeida et al. 2020; Chala et al. 2022; Cummings et al. 2017; Fullen et al. 2022; Herman et al. 2023; Hush et al. 2009; Martin et al. 2018; Mathew et al. 2013; Serbic and Pincus 2013; Snelgrove et al. 2013; M. Stensland 2021a, 2021b; M. L. Stensland and Sanders 2018a, 2018b, 2018c). A detailed summary of the publications included in the TLR is presented in Table 1.

**FIGURE 1:**
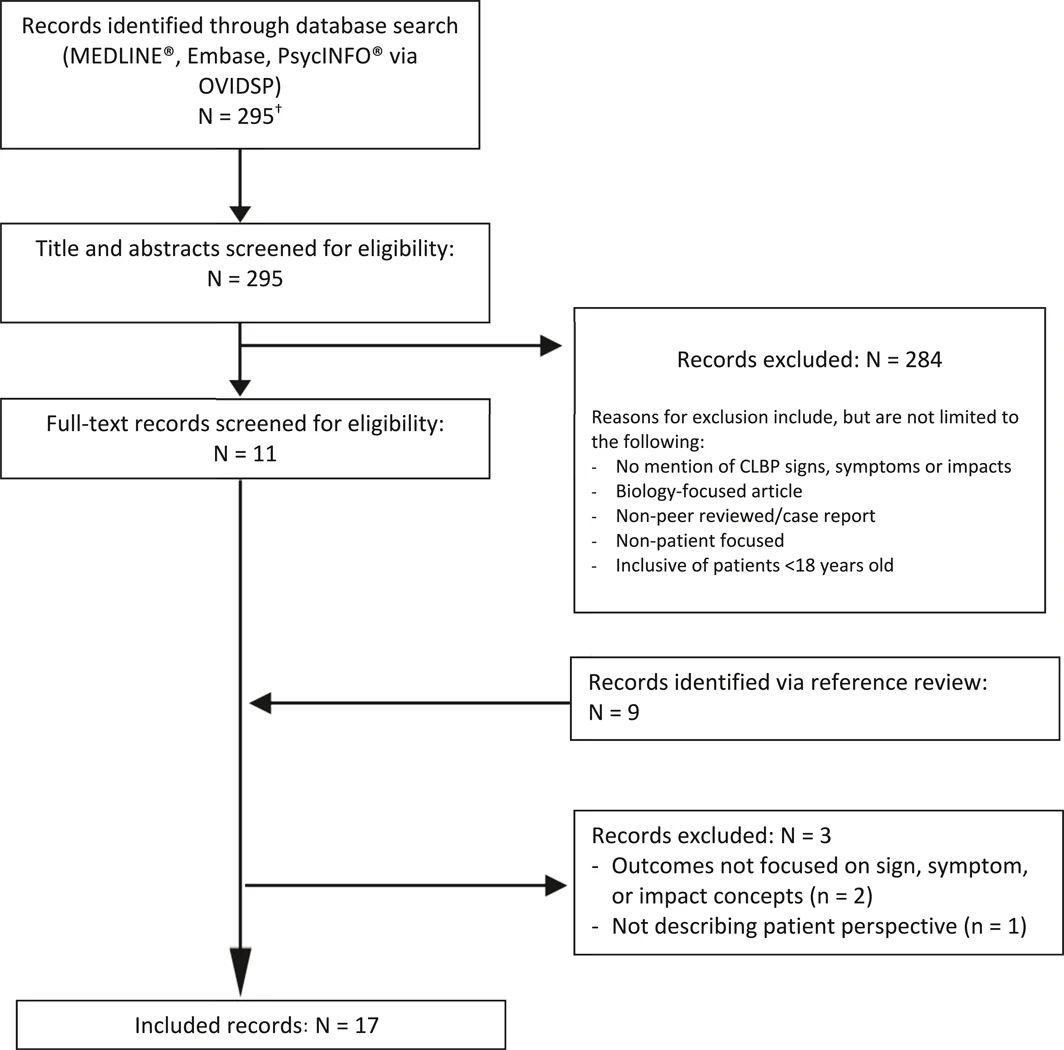
PRISMA diagram of selected articles. PRISMA = Preferred Reporting Items for Systematic Reviews and Meta‐analyses. ^†^All duplicates identified through the database search were removed by OVID and cannot be reported separately.

**TABLE 1:** Publications included in the TLR.

Reference	Study population	Aim of study	Data collection method	Completed analyses relevant to TLR
Bailly et al. (2015) The impact of chronic low back pain is partly related to loss of social role: A qualitative study	Study country: France Eligibility criteria summary: Sub‐acute or chronic nonspecific low back pain, had not received back surgery in the 3 months prior to study participation *N* = 25 participants (*n* = 14 women, *n* = 11 men) Age (years): range = 25–81	To better understand experiences in relation with participant, and social difficulties of patients living with chronic LBP, with a focus on impact on relationships with family, friends and work colleagues.” (Bailly et al. 2015, 437)	Qualitative focus groups	Thematic analysis
Bushnell et al. (2018) Mixed‐methods development of a new patient‐reported outcome instrument for chronic low back pain: part 2—The Patient Assessment for Low Back Pain–Impacts (PAL‐I)	Study countries: United States, Germany, United Kingdom Eligibility criteria summary: Confirmation of clinical diagnosis and current CLBP, duration of CLBP, pain intensity, absence of recent low back surgery or planned low back surgery in the next 30 days, and recent epidural injections or spinal cord stimulation therapy ◦ Participants with a ≥ 4 on a 0‐ to 10‐point numeric rating scale (NRS; moderate‐to‐severe pain) participated in interviews as the study targeted representation of patients with neuropathic CLBP Concept elicitation interview cohort: ◦ *N* = 43 (*n* = 23 female, *n* = 20 male) ◦ Age (years): Mean (m) = 43.6, range = 21–73 Initial cognitive interview cohort: ◦ *N* = 30 (*n* = 17 female, *n* = 13 male) ◦ Age (years): *m* = 43.6, range = 20–77 Cognitive interviews to evaluate modifications cohort: ◦ *N* = 16 (*n* = 8 female, *n* = 8 male) ◦ Age (years): *m* = 47.2, range = 30–78 Cognitive interviews to evaluate the electronic version cohort: ◦ *N* = 8 (*n* = 4 female, *n* = 4 male) ◦ Age (years): *m* = 41.5, range = 29–60 Study describes additional cohorts to achieve additional research objectives	To ‘describe the qualitative and quantitative development and preliminary validation of the Patient Assessment for Low Back Pain–Impacts (PAL‐I), a patient‐reported outcome measure for use in chronic low back pain (cLBP) clinical trials.’ (Bushnell et al. 2018, 2066)	Qualitative interviews Instrument administration (PAL‐S and PAL‐I)	Saturation analysis Thematic concept analysis Rasch measurement theory Classical test theory
Calmon Almeida et al. (2020) Do the commonly used standard questionnaires measure what is of concern to patients with low back pain?	Study country: Brazil Eligibility criteria summary: Clinical diagnosis of CLBP *N* = 27 (19 women, 8 men) Age (years): mean = 55.2 years old	To ‘evaluate whether questionnaires (i.e. the International Classification of Functioning, the Roland‐Morris Disability Questionnaire, the Quebec Back Pain Disability Scale, and the Oswestry Disability Index) identified all the self‐reported patient outcomes raised in focus groups.’ (Calmon Almeida et al. 2020, 1313)	Instrument administration (Roland‐Morris Disability Questionnaire) Qualitative interviews	Thematic analysis
Chala et al. (2022) An interpretive phenomenological analysis of living with chronic low back pain in Ethiopia	Study country: Ethiopia Eligibility criteria: Adults with a diagnosis of CLBP for at least ≥ 3 months who receive care at the university hospital *N* = 15 (10 women, 5 men) Age (years): range = 19–66	‘To explore the lived experiences of people with chronic low back pain in Ethiopia.’ (Chala et al. 2022, 4031)	Qualitative interviews	Interpretative phenomenological analysis
Cummings et al. (2017) Self‐efficacy and paradoxical dependence in chronic back pain: A qualitative analysis	Study country: United States Eligibility criteria: Having repeated episodes of CLBP over a period of at least 1 year *N* = 20 (16 women, 4 men) Age (years): range = 29–78	‘Patients with chronic back pain encounter considerable psychological and social challenges. In particular, the ever‐evolving relationship between chronic back pain, sense of self‐efficacy, and perceived role in interpersonal relationships merits further investigation.’ (Cummings et al. 2017, 251)	Qualitative interviews	Thematic analysis
Fullen et al. (2022) Management of chronic low back pain and the impact on patients' personal and professional lives: Results from an international patient survey	Study countries: Australia, Belgium, Finland, France, Germany, Israel, Italy, Netherlands, Norway, Romania, Russia, Spain, Sweden, Switzerland, Turkey, and the United Kingdom Eligibility criteria: Experience of CLBP over the past 12 weeks and clinician diagnosis *N* = 9642 (46.0% women, 54.0% men) Age (years): mean = 51	‘To investigate the impact of chronic low back pain on patients' personal and professional lives, and management strategies applied to treat CLBP.’ (Fullen et al. 2022, 436)	Survey	Crosstabs analysis Frequency analysis
Herman et al. (2023) Definitions of chronic low back pain from a scoping review, and analyses of narratives and self‐reported health of adults with low back pain	Study countries: Not specified Eligibility criteria: Adults with a diagnosis of CLBP for at least ≥ 3 months *N* = 1482 (48.1% women, 51.3% men) Age (years): mean = 39.6 years old	‘To [1] …examine the published literature for recent randomized controlled trials (RCTs) of patients with CLBP to understand how CLBP is defined, [2] …examine the statements made win a recent survey of individuals with LBP about how they define their pain as chronic, [3] …examine the frequency and overlap between four chronicity definitions applied to general LBP sample and how their demographics, pain management behaviours, and outcomes differ by the definition of CLBP used.’ (Herman et al. 2023, 404)	Scoping review of previously published CLBP literature (236 articles reviewed) Survey	Content analysis Description analysis Frequency analysis
Hush et al. (2009) Recovery: What does this mean to patients with low back pain?	Study country: Australia Eligibility criteria: CLBP pain patients (recovered and not recovered) with no history of specific spinal pathology *N* = 36 (15 women, 21 men) Age (years): mean = 41, range (31–59)	‘To explore patients' perceptions of recovery from low back pain, about which little is known.’ (Hush et al. 2009, 124)	Qualitative focus groups	Thematic analysis
Martin et al. (2018) Mixed‐methods development of a new patient‐reported outcome instrument for chronic low back pain: part 1‐the Patient Assessment for Low Back Pain—Symptoms (PAL‐S)	See Bushnell et al. 2018 above	To ‘describe the qualitative and quantitative development and preliminary validation of the Patient Assessment for Low Back Pain–Symptoms (PAL‐S), a patient‐reported outcome measure for use in chronic low back pain (cLBP) clinical trials.’ (Martin et al. 2018, 1045)	Instrument administration (PAL‐S) Qualitative interviews	Saturation analysis Thematic concept analysis Rasch measurement theory Classical test theory
Mathew et al. (2013) Backing up the stories: The psychological and social costs of chronic low‐back pain	Study country: Australia Eligibility criteria: Diagnosis of CLBP with or without leg pain for > 3 months *N* = 30 (sex not specified) Age (years): Not specified	‘To analyse and evaluate the personal and psychosocial costs of chronic low‐back pain.’ (Mathew et al. 2013, 29)	Instrument administration (Short Form 36 Health Survey, the Depression Anxiety Stress Scale)	*t‐*tests analysis Frequency analyses
Serbic and Pincus (2013) Chasing the ghosts: The impact of diagnostic labelling on self‐management and pain‐related guilt in chronic low back pain patients	Study country: United Kingdom Eligibility criteria: CLBP patients seeking treatment with a pain duration of at least 3 months *N* = 20 (14 women, 6 men) Age (years): mean = 46.4, range = 54–76	‘To examine CLBP patients' understanding, feelings, and behaviour in response to their diagnostic labels.’ (Serbic and Pincus 2013, 25)	Qualitative interviews	Thematic analysis
Snelgrove et al. (2013) A longitudinal study of patients' experiences of chronic low back pain using interpretative phenomenological analysis: Changes and consistencies	Study country: United Kingdom Eligibility criteria: Unrelieved, benign CLBP with no definitive diagnosis and increasing distress and disability *N* = 10 (6 women, 4 men) Age (years): range = 40–76	To ‘[explore] the “lived experiences” of patients with chronic low back pain (CLBP) in the United Kingdom.’ (Snelgrove et al. 2013, 121)	Qualitative interviews	Thematic analysis
M. Stensland (2021a) “If you don't keep going, you're gonna die”: Helplessness and perseverance among older adults living with chronic low back pain	Study country: United States Eligibility criteria: Pain clinic patient, 65 years old or older, with reported of CLBP for at least 3 months *N* = 21 (13 women, 8 men) Age (years): range = 66–83	‘To understand older adults' lived CLBP experience.’ (M. Stensland 2021a, 907)	Qualitative interviews	Thematic analysis
M. Stensland (2021b) Managing the incurable: older pain clinic patients' experiences of managing treatment‐resistant chronic low back pain		‘To examine how older adults experience pain management approaches for CLBP.’ (M. Stensland 2021b, 405)		
M. L. Stensland and Sanders (2018a) “It has changed my whole life”: The systemic implications of chronic low back pain among older adults		‘To understand how older adults experience CLBP and its impacts on the functioning of adults.’ (M. L. Stensland and Sanders 2018a, 129)		
M. L. Stensland and Sanders (2018b) Living a life full of pain: Older pain clinic patients' experience of living with chronic low back pain		‘To understand older adults' lived CLBP experience.’ (M. L. Stensland and Sanders 2018b, 1434)		
M. L. Stensland and Sanders (2018c) Not so golden after all: The complexities of chronic low back pain in older adulthood		‘To understand how chronic low back pain (CLBP) impacts key ageing concept such as retirement, housing, health, and independence.’ (M. L. Stensland and Sanders 2018c, 923)		

Seven key symptom concepts related to CLBP, including back pain, fatigue, body pain, cramping, numbness, stiffness, and muscle weakness, were reported in multiple studies from the TLR. These symptoms were reported to impact patients' lives across multiple domains. Commonly reported HRQoL impacts were on emotional functioning (e.g., feeling depressed), physical functioning (e.g., difficulty walking), work, and use of adaptive behaviours (e.g., reliance on assistive devices).

### 4.2 Qualitative Patient Interviews

Table 2 shows the demographic and health information of the 30 participants. Sample diversity based on sex, race, ethnicity, and education level was achieved. Conceptual saturation analysis showed that nearly all symptom concepts (with the exception of one) and all HRQoL impact domains were reported spontaneously by participants in the first 75.0% of interviews. Accordingly, the key symptoms and HRQoL impact domains related to the experience of CLBP were adequately identified during the interviews.

**TABLE 2:** Qualitative interview participant demographic and health information.

Characteristic	Total sample (*N* = 30) *n* (%)
Age^a^	
Range	22.5–86.0
Mean (standard deviation)	48.2 (16.1)
Sex (assigned at birth)^a^	
Female	17 (56.7%)
Male	13 (43.3%)
Race^a^	
White	19 (63.3%)
Black or African American	8 (26.7%)
Asian	3 (10.0%)
Ethnicity^a^	
Not of hispanic, latino/*a*/*x*,^b^ or Spanish origin	24 (80.0%)
Mexican, Mexican American, Chicano/*a*/*x* ^b^	5 (16.7%)
Prefer not to say	1 (3.3%)
Current work status^a^	
Working full‐time	20 (66.7%)
Retired	5 (16.7%)
Out of work for 1 year or more	2 (6.7%)
Working part‐time	2 (6.7%)
Homemaker	1 (3.3%)
Highest level of education^a^	
Some high school or less	3 (10.0%)
High school diploma/GED	11 (36.7%)
Some college/certificate programme	3 (10.0%)
Completed certificate programme/associate degree (2‐year)	6 (20.0%)
Undergraduate degree	6 (20.0%)
Post‐graduate degree	1 (3.3%)
Annual household income^a^	
Under $25,000	2 (6.7%)
$25,000–$49,999	7 (23.3%)
$50,000–$74,999	7 (23.3%)
$75,000–$99,999	6 (20.0%)
$100,000 and over	1 (3.3%)
Prefer not to answer	7 (23.3%)
Body mass index (BMI)^a^ ^,^ ^c^	
≥ 30.0	15 (50.0%)
27.0 to 29.9	6 (20.0%)
< 27.0	6 (20.0%)
Not reported	3 (10.0%)
Patient self‐reported rating of overall health^a^	
Excellent	2 (6.7%)
Very good	5 (16.7%)
Good	16 (53.3%)
Fair	7 (23.3%)
Other health conditions^a^ ^,^ ^d^	
None	12 (40.0%)
Asthma	5 (16.7%)
Diabetes (Type II)	5 (16.7%)
High blood pressure	5 (16.7%)
Anxiety	4 (13.3%)
High cholesterol	4 (13.3%)
Migraine headaches	2 (6.7%)
Autoimmune disease	1 (3.3%)
Breast cancer	1 (3.3%)
Chronic obstructive pulmonary disease, emphysema, chronic bronchitis	1 (3.3%)
Kidney disease	1 (3.3%)
Liver disease	1 (3.3%)
Osteoarthritis	1 (3.3%)
Other (thyroid condition)	1 (3.3%)
Duration of CLBP^a^	
1–5 years	17 (56.7%)
6–10 years	6 (20.0%)
More than 10 years	7 (23.3%)
Medication used in the past to treat CLBP (not mutually exclusive)^a^	
Nonsteroidal anti‐inflammatory drugs (e.g., meloxicam, ibuprofen)	21 (70.0%)
Muscle relaxants (e.g., cyclobenzaprine, zanaflex, diazepam)	17 (56.7%)
Non‐opioid analgesics (e.g., aspirin, acetaminophen, paracetamol)	17 (56.7%)
Prescription opioids (e.g., morphine, codeine, hydrocodone, oxycodone)	7 (23.3%)
Gabapentinoids (e.g., gabapentin, pregabalin)	5 (16.7%)
Steroids (e.g., prednisone, triamcinolone)	1 (3.3%)
Current medication used to treat CLBP (not mutually exclusive)^a^	
Nonsteroidal anti‐inflammatory drugs (e.g., meloxicam, ibuprofen)	20 (66.7%)
Non‐opioid analgesics (e.g., aspirin, acetaminophen, paracetamol)	17 (56.7%)
Muscle relaxants (e.g., cyclobenzaprine, zanaflex, diazepam)	8 (26.7%)
Prescription opioids (e.g., morphine, codeine, hydrocodone, oxycodone)	6 (20.0%)
Gabapentinoids (e.g., gabapentin, pregabalin)	3 (10.0%)
Steroids (e.g., prednisone, triamcinolone)	1 (3.3%)
Patient self‐reported pain severity: Over the past 7 days, how would you rate the usual level of your lower back pain? (0—No pain at all—10—Worst pain possible)^a^ ^,^ ^e^	
4	4 (13.3%)
5	3 (10.0%)
6	8 (26.7%)
7	3 (10.0%)
8	9 (30.0%)
9	2 (6.7%)
10 (Worst possible lower back pain)	1 (3.3%)
Patient self‐reported pain frequency: Over the past 7 days, how often did you experience lower back pain? (0–7 days)^a^ ^,^ ^e^	
4 days	4 (13.3%)
5 days	6 (20.0%)
6 days	4 (13.3%)
7 days	16 (53.3%)

^a^ Patient‐reported data.

^b^ Latinx/Chicanx is a gender‐neutral or nonbinary alternative to Latino/Chicano or Latina/Chicana.

^c^ Height and weight data collection and BMI calculation were completed post hoc to the interview study. Approximately 1 year after the interviews, participants were recontacted and asked to report their height and estimate their weight at the time of their interview.

^d^ Other health conditions were inclusive of past and current health conditions.

^e^ Participants were required to have pain severity of 4 or greater and pain duration for four or more days as part of inclusion criteria.

#### 4.2.1 Patient Experience of Pain and Other Symptoms

All participants spontaneously reported back pain, most frequently describing it as ‘sharp’ (*n* = 11/30), ‘dull’ (*n* = 9/30), ‘ache’ (*n* = 7/30), and ‘stabbing’ (*n* = 5/30). They described heterogeneous experiences of back pain in terms of severity, frequency, duration, and location. Participants described their back pain as ‘excruciating’ and ‘unpredictable,’ with one participant specifying their experience as ‘It's like someone is taking a fire poker and…. literally going across that lower band of my back’. The frequency ranged from multiple times a day to a couple of times a month, while the duration ranged from ‘a few minutes’ to ‘all day’, depending on the type of pain. Participants also reported their back pain radiating to other parts of their body, including their leg, ankle/toes, and shoulders/neck.

Seven key symptom concepts associated with CLBP were reported by participants, including muscle spasms, numbness, stiffness, tingling, fatigue, headache, and weakness. Participants described that these symptoms commonly occurred with pain.

#### 4.2.2 Patient Experience of HRQoL Impacts

Most participants spontaneously described that their physical (*n* = 29/30) and emotional (*n* = 28/30) functioning were negatively impacted by their CLBP. Impacts on walking (*n* = 20/30), limited participation in social activities (*n* = 22/30), frustration (*n* = 20/30), and difficulty staying asleep (*n* = 19/30) were most frequently reported. Participants most frequently reported that impacts on physical functioning, sleep, emotional functioning, social functioning, and household chores/responsibilities were the most bothersome or most important to improve.

Ultimately, participants described wide‐ranging negative impacts from their CLBP that compound to affect many areas of their lives as a result, often causing them to rearrange their plans or approach to getting through their daily responsibilities. For example, one participant explained:

Almost 40% of my daily life is definitely impacted ‘cause certain things’, it'll take me longer to do because I'll think about what I'm about to do, if I'm gonna do, you know—do grocery shopping. Whatever I do, I think first, I‐I watch my movement, everything, because I don't wanna have a bad day. I don't wanna have, like, two bad days because I overexerted my back, meaning, you know.… Say, for instance, if I'm gonna do something today, meaning go grocery shopping and maybe even do laundry …. I'm only gonna do one load, very light.… I said everything that I do, you just gotta be careful.

Another participant described that their CLBP impacts ‘mundane’ activities, such as:

…walking at times or they’re er, just doing mundane things like maybe tying your shoe or er, um, or if I have to lift something, obviously I’m not gonna lift something that’s heavy. But those, you know, the sporadic things you have to do during a day … that are really mundane. People don’t think about it. Maybe I have a problem with it that day.

Lastly, one participant shared how their CLBP limits them:

I can’t walk for very long, um.… you know, when we’re going through a store or something, to do grocery shopping, it—I can’t get, be in a store, like I can’t just, just walk around and look at everything because that takes too much of a toll on me. I have to then take breaks and sit, um, because I can’t do things for long periods of time.… I can’t—I can’t walk for long periods, I can’t—There, there’s so many limitations. It, it, I mean everything you think of what you do in a normal day, I can’t do that.

### 4.3 Merging Patient‐Experience Data and Development of the Conceptual Model

The conceptual model displays the patient experience of CLBP based on the findings of this work, incorporating 16 symptoms and 120 HRQoL impacts (across 18 HRQoL domains) associated with CLBP (Figure 2). The majority of the concepts were merged because they were elicited in both the TLR findings and the patient interviews. However, some concepts were uniquely identified in each source, as illustrated in Figure 2. Table 3 presents the most frequently reported symptoms (i.e., reported by ≥ 20.0% of participants or identified in ≥ 20.0% of articles) and HRQoL impact domains and concepts (i.e., reported by ≥ 20.0% of participants or identified in ≥ 20.0% of articles, when applicable) in the qualitative interviews and TLR.

**FIGURE 2:**
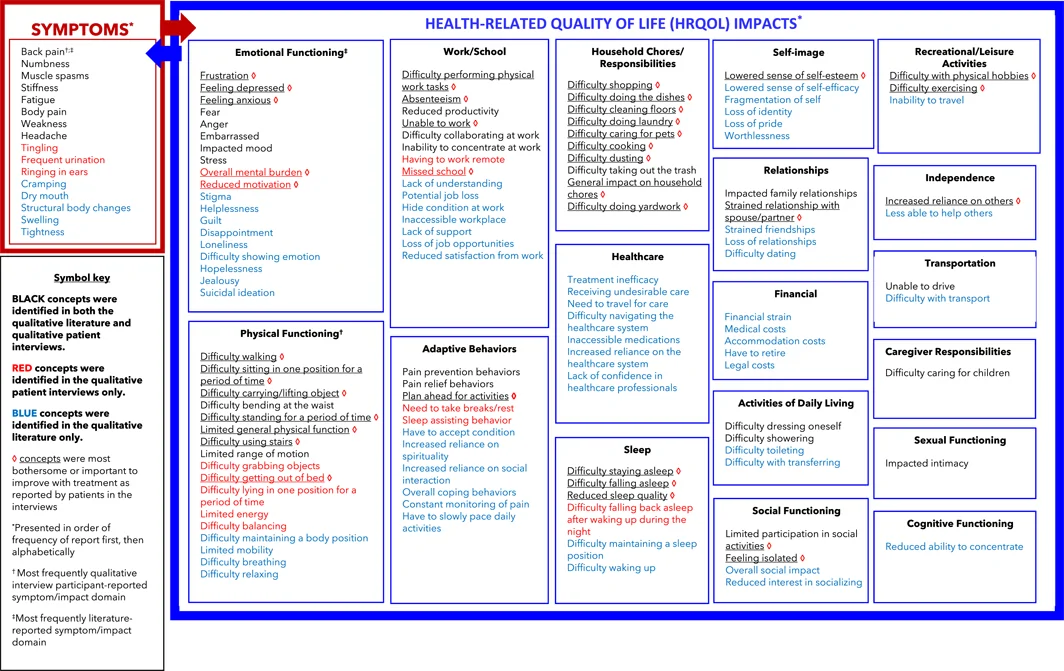
Patient‐centered conceptual model.

**TABLE 3:** Frequently reported^a^ CLBP symptoms and impact concepts from the TLR and qualitative interviews.

CLBP‐related domain or concept	Frequency of identification in TLR articles *N* = 17 *n* (%)	Frequency of participant report during qualitative interviews *N* = 30 *n* (%)
Symptoms		
Back pain	15 (88.2%)^b^	30 (100.0%)
Numbness	2 (11.8%)	7 (23.3%)
Muscle spasms	1 (5.9%)	7 (23.3%)
Fatigue	5 (29.4%)	2 (6.7%)
HRQoL impact domains and concepts		
Physical functioning	11/17 (64.7%)	29/30 (96.7%)
Difficulty walking^c^ ^,^ ^d^	7 (41.2%)	20 (66.7%)
Difficulty sitting in one position for a period of time^c^	4 (23.5%)	17 (56.7%)
Difficulty lifting/carrying objects^c^ ^,^ ^d^	3 (17.6%)	16 (53.3%)
Difficulty bending at the waist	3 (17.6%)	12 (40.0%)
Difficulty standing for a period of time	4 (23.5%)	9 (30.0%)
Limited general physical function	1 (5.9%)	9 (30.0%)
Difficulty using stairs	3 (17.6%)	6 (20.0%)
Emotional functioning	14/17 (82.4%)	17/30 (56.7%)
Frustration^d^	8 (47.1%)	20 (66.7%)
Feeling depressed^c^ ^,^ ^d^	11 (64.7%)	7 (23.3%)
Overall mental burden	—	8 (26.7%)
Reduced motivation^d^	—	6 (20.0%)
Feeling anxious^d^	10 (58.8%)	1 (3.3%)
Fear	7 (41.2%)	4 (13.3%)
Stigma	6 (35.3%)	—
Helplessness	5 (29.4%)	—
Guilt	4 (23.5%)	—
Adaptive behaviours	8/17 (47.1%)	26/30 (86.7%)
Pain prevention behaviours	10 (58.8%)	18 (60.0%)
Pain relief behaviours	4 (23.5%)	13 (43.3%)
Need to take breaks or rest	1 (5.9%)	10 (33.3%)
Have to accept condition	4 (23.5%)	—
Sleep	6/17 (35.3%)	25/30 (83.3%)
Difficulty staying asleep^c^ ^,^ ^d^	6 (35.3%)	19 (63.3%)
Difficulty falling asleep^c^ ^,^ ^d^	1 (5.9%)	11 (36.7%)
Reduced sleep quality^c^ ^,^ ^d^	1 (5.9%)	7 (23.3%)
Social functioning	9/17 (52.9%)	23/30 (76.6%)
Limited participation in social activities^c^ ^,^ ^d^	9 (52.9%)	23 (76.7%)
Feeling isolated^d^	5 (29.4%)	3 (10.0%)
Overall social impact	5 (29.4%)	—
Household chores/responsibilities	9/17 (52.9%)	20/30 (66.7%)
Difficulty shopping^d^	7 (41.2%)	8 (26.7%)
Difficulty cleaning floors^c^ ^,^ ^d^	7 (41.2%)	6 (20.0%)
Difficulty doing the dishes^c^	7 (41.2%)	6 (20.0%)
Work/school	9/17 (52.9%)	14/30 (46.7%)
Difficulty performing physical work tasks^c^ ^,^ ^d^	4 (23.5%)	7 (23.3%)
Reduced productivity	5 (29.4%)	1 (3.3%)

Abbreviations: CLBP = chronic low back pain; HRQoL = health‐related quality of life; TLR = targeted literature review.

^a^ Reported by ≥ 20.0% of participants or identified in ≥ 20.0% of articles.

^b^ Two articles included in the TLR did not report back pain as a symptom as they focused on CLBP impacts and pain recovery.

^c^ Concept was reported as most important to improve by participants in interviews.

^d^ Concept was reported as most bothersome by participants in interviews.

The cardinal symptom of CLBP, pain, was reported and described by all participants in the qualitative interviews and in more than 80.0% (i.e., 15 out of 17 TLR articles) of articles in the TLR. Two articles (Bushnell et al. 2018; Hush et al. 2009) that did not describe the experience of back pain were focused on the exploration of pain‐related impacts or recovery. Descriptions of pain quality were similar in the TLR and qualitative interviews, including terms such as ‘sharp’ (Herman et al. 2023; Martin et al. 2018), ‘dull’ (Martin et al. 2018), ‘ache’ (Hush et al. 2009; Martin et al. 2018; M. L. Stensland and Sanders 2018b), and ‘stabbing’ (Martin et al. 2018). Several co‐occurring symptoms of CLBP were reported (reported by 60.0% of qualitative interview patients and in 47.1% of TLR articles), including numbness, muscle spasms, stiffness, tingling, and fatigue.

Findings showed that CLBP negatively impacts multiple domains of HRQoL. The most frequently reported HRQoL impacts experienced by patients were related to physical functioning (e.g., difficulty walking, difficulty sitting for long periods of time), emotional functioning (e.g., frustration, feeling depressed), adaptive behaviours (e.g., pain prevention behaviours such as avoiding carrying heavy objects, only sitting in supportive chairs, and wearing supportive shoes to prevent triggering pain or reduce the amount of pain they experience), sleep (e.g., difficulty staying asleep), household chores/responsibilities (e.g., difficulty shopping, difficulty cleaning floors), work/school (e.g., difficulty completing physical work tasks), and social functioning (e.g., limited participation in social activities).

Table 4 presents example patient experience quotes from the TLR and patient interviews for symptoms and key HRQoL domains. These quotations describe experiences of bothersome and burdensome concepts within the most frequently reported domains reported above in Table 3.

**TABLE 4:** Participant quotes from the TLR and patient interviews.

Domain	Example patient quotes from the TLR	Participant quotes from qualitative interviews
Back pain	‘It just kills you. You know, it just‐god, it just takes you down, and I can say mentally and physically, it just wears you out. It wears you down. It wears on you all the time.’ (M. L. Stensland and Sanders 2018b, 1439) ‘I have pain regularly; it is almost always there, sometimes severely, and sometimes less severely, but I live with it constantly. I can barely remember when it wasn't like this.’ (Herman et al. 2023, 406)	**Participant 1:** ‘I have lower back pain across the right and left side of the ba—lower back.… it starts around in the middle of the back and goes down around the hip, down the leg, around the knee, and all the way down my leg to the ankle.… On both sides, um, it gets extremely painful at times, and then there are days when it isn't.… it can be like a burning sensation.… Sometimes it's a—A sharp pain. Sometimes it's just a dull ache.… I can have just a dull ache all day, where I can be fine and all of a sudden have a sharp pain or an ache in the back, and then it will be bad all day.… Or, as I say, there are days when they have both, you know?’ **Participant 2:** ‘INTERVIEWER: Can you … help me understand what your lower back pain is like for you? PARTICIPANT: Well, it's er, acute sharpness er, pain, er, in the lower back region there.… I would say er, at times it could, without medication, it could be excruciating**.**… obviously you have good days and then bad days.… activity wise, physical activities are very tough to handle … more than any other activities. Um, but I try to suck it up and er, carry on.’
Physical functioning	‘“It's very hard to walk. Before, in my job, I used to clean 19,000 square feet a day. Seventeen flights of stairs. I used to run up and down a lot of them. I used to be able to carry fifty pounds up those stairs. I've got a ten‐pound weight limit right now. For a while there, making it up one flight of stairs was very difficult. I used to stub my toe trying to lift my leg. So that's fairly tough. It feels like losing a hand.’ (Cummings et al. 2017, 255)	**Participant 3:** ‘I'm back into that mode where I'm trying to… to put all my strength into that actual movement of going from a sitting position to a standing position, or‐or going from a kneeling position to a standing position.… so, it… again, depending on the severity of the‐the pain and the discomfort, it‐it can be quite difficult to accomplish that feat. But again, it's‐it's something that I do.… but it just takes a‐a longer period of time.… and it… like I said, the back is the back, it is more of a centre focus in‐in the human body.… than people realise.… until they start getting back pain.… then it's affected in everything you do.… going to the bathroom, showering, eating, sitting, driving, you know, this… you're… it… sleeping.… it im‐… it impacts your‐your daily life. So, I would definitely, if there was some sort of treatment that could remedy that completely.… yeah, it would be for everything.’
Emotional functioning	‘It was very, very tough [being in so much pain and not being able to explain to herself and other people what really caused it], it was very depressing; I ended up extremely depressed.’ (Serbic and Pincus 2013, 30)	**Participant 4:** ‘Um, it can be very overwhelming. Um, I have a job where I stand up, so someti—sometimes that can be a 10. I have to be mentally prepared to, um—It's like a challenge for my body, for my back and, um, it's really something—I have to be—you have to be prepared, and I usually just start to stretch, um, so I have to, like, um, always move my body as much as I can.… Um, but I have hot back heating pad that I try to always sleep in at night, but, um.… It is a task, for sure.’
Adaptive behaviours	‘Before I go places I make sure that they're accessible and any more, almost everything is or that <spouse> is going with me because like he can carry my stroller—it folds and he can carry my stroller upstairs. If there's railings, I can get up the stairs, you know. When I go into someone's house, I look for the hardest, highest chair to sit on and if there isn't any, I sit on my stroller.’ (M. Stensland 2021b, 1440)	**Participant 5:** ‘I've kinda gotten into a routine. No, no. I've gotten into a routine where I've kinda taken away all—You know, all the things that—that, um, affect it negatively …. Uh, and spent more—spent more time at the gym in the pool, uh, stretching, yoga. Uh, I've lost 80 pounds over the last year, which has helped immensely.… although, there's still—there's still limited range of motion.… Um, it just, uh—Yeah, it required a whole kinda reset of my lifestyle …. To be honest with you.… INTERVIEWER: Has your, um, back pain ever affected your ability to sit?.… PARTICIPANT: Yes, it does. I can't sit more than maybe 20 minutes, 25 minutes.… Yeah, I have—I‐I can't sit in soft furniture, you know, the overstuffed things, the.… the recliners and la‐Z‐boys people have, I don't—I have some wingback chairs that seem to help and a rocking chair, a Kennedy rocker that I use for most of my sitting when I do sit. A lotta times, if I'm working at my desk, I stand.… So, that—That's led to a whole different, you know, a‐a‐approaches. Uh, for shoes, I have to make sure that they—I kinda try to replace them, you know, every 6 months or so, just because, you know, the arches fall over time and I can tell. I can tell when the shoes are worn out.’
Sleep	‘You're never free of pain. I would love to have no pain just for a few hours, especially at night. It is dreadful. You are unable to go into bed and lie down like normal people and go to sleep.’ (Snelgrove et al. 2013, 127)	**Participant 6: ‘**Well, it's just, I find that if I don't, er, if I have trouble getting to sleep or staying asleep, then of course, that affects, you know, sets the whole tone for the next day…. Because then if you're like, okay, I didn't sleep well, I don't feel well, so then it, kinda puts a damper on the whole next day, you know, and I kinda have to plan around that not feeling good, or not being able to do what I want to do.’
Social functioning	‘For example, when we go on holidays, my wife has to carry our luggage. Everyone is looking at me, I’m very muscular and people don’t understand why I’m not carrying them. In the subway, women ask me to help them carry their strollers and I have to say “sorry but I can't help you.”’ (Bailly et al. 2015, 439) ‘It is damaging when your kids cannot get what they deserve from you. One day, my daughter asked me, “papa why don't you walk me to the school like other parents of my classmate friends?”’ (Chala et al. 2022, 4036)	**Participant 7:** ‘I think the most bothersome one is, um, impacting my social, um, because if I have a plan to do something and then I'm just uncomfortable, you know, it's—and I cancel at the last minute…. It impacts other people's time and it impacts my time, and I'm missing out on events I would normally go to…. That's probably the most impactful for me.’ **Participant 8:** ‘…this might sound a little shallow but, my ability to be able to like, do things with my family is probably the most impactful. I mean, I may be able to live without sweeping the floor but when everyone… wants to do an activity and I can't be present, or I can't go, I feel like I'm missing out on building great memories and moments with my family because I'm always in pain.’
Household chores/responsibilities	‘I feel bad for not assisting my parents with some chores. You know…you cannot do anything when the pain prevents you from getting up from the chair to move.’ (Chala et al. 2022, 4036)	**Participant 1:** ‘INTERVIEWER: Um, which of those kind of impacts, um, of your lower back pain are the most bothersome to you? PARTICIPANT: Oh, well, trying to clean the house is very…. Hard. Um—and, as I say, I have a lot of stairs, so that…. Is also a problem. Uh, I manage but, you know, it's difficult because it does cause pain sometimes. Um—So, I think cleaning and going up and down the stairs is probably, you know, the biggest thing that I have…. That bothers me the most, yeah.’
Work/school	‘Some days, due to the pain, I just cannot get out of bed though I want to. Getting my employer to understand this appears to be an impossible task. Work is planned, independently of our condition, and we have to do it.’ (Bailly et al. 2015, 439)	**Participant 10:** ‘INTERVIEWER: …How about your, like, ability to go to work or school or, um, anything like that, do you feel like your back pain has impacted that at all? PARTICIPANT: Yes, absolutely. INTERVIEWER: Okay. How so? PARTICIPANT: Impact—It's—Well, I, uh—I retired early because of it, [laughs].’

## 5 Discussion

This study explored the patient perspective of the burden of non‐specific CLBP through a TLR and qualitative interviews. The TLR identified contemporary studies describing the symptoms and HRQoL impacts of CLBP, while the interviews were conducted to build on this knowledge and validate the most relevant and important concepts for patients with CLBP focussing on their descriptions of back pain and how this impacts their daily life. Findings were merged into a conceptual model to build a deeper understanding of the patient experience, which is necessary to inform the development of COA measurement strategies for patient‐focused drug development in line with industry standards. Findings highlight that CLBP is a burdensome condition with a myriad of physical symptoms and experiences that interfere with patients' daily lives.

The majority of the publications in the TLR and all interview participants reported experiencing back pain. However, patients' lived experiences of pain varied in severity, frequency, duration, and radiating location, highlighting the heterogeneity of patients' experiences. CLBP measurement strategies may benefit from the use of a daily pain assessment that captures these multiple dimensions and impacts of pain, as the pain experience is ‘continuous, overlapping, and dynamic’ and pain status can transition over time (i.e., pain can be newly onset, worsening, improving, resolving, or maintained (Eccleston et al. 2023).

Beyond back pain, seven co‐occurring symptoms were identified across both sources: numbness, muscle spasms, stiffness, fatigue, body pain, weakness, and headache. While 60.0% of the qualitative interview sample and 47.1% of TLR articles reported at least one of these co‐occurring symptoms, each symptom was reported by less than 30.0% of the patients or articles, highlighting the heterogeneity of patient experience. Some symptoms were infrequently reported and unique to specific research activities. For example, cramping, dry mouth, structural body changes, swelling, and tightness were only identified in the TLR, while tingling, frequent urination, and ringing in ears were uniquely reported in qualitative interviews. Given this variability, PRO measurement strategies should ideally be developed based on input from the specific target patient population for the treatment to ensure the most relevant and important concepts are measured.

Results suggest that CLBP has broad impacts on patients' lives, including negatively impacting sleep, work/school, household chores/responsibilities, and overall physical, emotional, and social functioning. This suggests that individuals with CLBP may often be experiencing high‐impact chronic pain, defined as ‘chronic pain that frequently limits life or work activities’ (J. Dahlhamer et al. 2018; J. M. Dahlhamer et al. 2016; Vaegter et al. 2023). The potential for CLBP to be experienced by patients as high‐impact chronic pain has implications for clinician and patient discussions and decision‐making related to treatment. The efficacy of treatments for CLBP should be evaluated for their ability to demonstrate a meaningful reduction in pain and pain interference with other areas of life. Treatment plans should be individualised to reflect patient priorities of treatment attributes, such as side effect tolerance versus magnitude of pain relief (Wilson et al. 2023).

This study showed that the impacts of CLBP on emotional functioning were particularly salient. In the qualitative interviews, participants reported frustration, feeling depressed, reduced motivation, and feeling anxious among the most bothersome and/or most important to improve, echoing the NIH Pain Consortium Task Force's recommendation that CLBP research assess depression and catastrophizing, as they are important to a wide range of patients with CLBP (Deyo et al. 2015). This highlights the need for comprehensive treatment options, which primarily address pain and also provide functional support to patients. For example, concurrently targeting pain and emotional dysregulation could serve as a model for future multi‐dimensional treatment approaches to pain management (Norman‐Nott et al. 2025).

The conceptual model developed as part of this study presents a comprehensive, non‐exhaustive synthesis; nevertheless, it provides a new presentation of the symptom and HRQoL impact experience of adults with CLBP as it presents integrated findings from a TLR and qualitative interviews to guide measurement strategy. Prior published conceptual models (Bailly et al. 2015; Calmon Almeida et al. 2020; Chala et al. 2022; Mathew et al. 2013; Serbic and Pincus 2013) differ in scope: some incorporate factors beyond symptoms and HRQoL impacts (e.g., non‐health‐related factors impacting CLBP experience (Calmon Almeida et al. 2020) and implications of a lack of clear diagnosis (Serbic and Pincus 2013) or present a narrower focus on specific themes (e.g., the social impact of CLBP (Bailly et al. 2015)). These models present findings from single data sources (e.g., focus groups or qualitative interviews with patients) rather than integrated findings across data sources, as in the present study. Confirming that key concepts of measurement for CLBP are consistent across multiple sources can help increase confidence in selecting these concepts for inclusion in a CLBP COA measurement strategy.

The patient‐centred conceptual model developed in this study can be utilised to assess the content validity of COAs and develop measurement strategies for future clinical trials for CLBP. Indeed, Ramasamy et al. (2017) conducted an evaluation of 13 PRO measures commonly used to assess CLBP symptoms and related HRQoL impacts, showing none of which fulfilled all criteria based on FDA guidance for use to support labelling claims (e.g., content validity, reliability, validity, ability to detect change) (Ramasamy et al. 2017). Thus, caution should be exercised when selecting an existing PRO measure to assess CLBP in a clinical trial setting and additional research to collect evidence of content validity (e.g., via cognitive patient interviews/pilot testing within the target patient population) or psychometric performance and interpretability of scores in the target patient population (e.g., using PRO data collected in a clinical trial) is recommended. The current conceptual model could be a useful resource for evaluating content validity for COAs; however, it is crucial to consider the overlap in patient populations when leveraging the published literature for evaluation of content validity. This work demonstrated the impactful yet heterogeneous patient experience of CLBP, which must be incorporated into PRO development/use and endpoint selection for each specific context of use.

The current findings complement and extend the foundational research standards established by the NIH Pain Consortium Task Force (Deyo et al. 2015). The conceptual model provides patient‐centred qualitative evidence that aligns with the Task Force's core outcome domains, while also providing expanded insight into the breadth of HRQoL impacts experienced by adults with CLBP. Continued alignment with established methodological standards supports cumulative knowledge‐building and enhances the comparability of PRO evidence across CLBP studies.

### 5.1 Strengths and Limitations

The conduct of qualitative patient interviews represents the key strength of this study: the patient perspective on their experience of CLBP. The interviews completed during this study collected data from a diverse and heterogeneous population, resulting in robust results that are representative of the population. The focused interview guide elicited patients' descriptions of how pain impacted their quality of life, which mitigates some risk of eliciting diluted quality of life impact data (e.g., related to other chronic diseases or conditions the sample experiences). While the sample size was limited to *N* = 30 participants, saturation of concept was demonstrated for nearly all symptom concepts (except ringing in ears), and all HRQoL impact domains, which provides confidence that the relevant and important concepts comprising the CLBP patient experience were reported within the interviews and that the conduct of additional interviews would not identify new, relevant CLBP concepts. An additional strength of this study is the conceptual model, inclusive of both literature‐based and patient‐reported data, which can be used to guide PRO measurement strategy and the selection of appropriate COAs as it summarises the meaningful aspects of health for this patient population.

This study has some limitations. CLBP is inclusive of a broad and heterogeneous patient population; thus, the characteristics of patients described in the TLR studies and of those who participated in qualitative interviews were not identical. The TLR was targeted and results were limited to work published between 2013 and 2023; thus, some publications may have been inadvertently missed during study identification. Participants in qualitative interviews were recruited only from clinical sites in the US, limiting generalisability of findings to patient populations outside of the US; however, TLR included studies conducted from 18 countries outside of the US. While the qualitative study implemented diversity targets, the sample lacked representation for some demographics (e.g., no Native American participants), which may affect the applicability of the conceptual model in some contexts of use; future research including typically underrepresented demographic characteristics should be conducted to explore the CLBP experience amongst diverse samples.

### 5.2 Conclusion

Given the impact of CLBP on patients, results from the research specify several symptom and HRQoL impact outcomes of interest for adults living with CLBP. The multidimensional pain experience and its myriad of impacts on daily life highlight the need to adequately measure the wide array of debilitating impacts on patients' lives. However, not all concepts were identified in both sources, due to variation in patient populations and research objectives across the TLR and qualitative interviews. The conceptual model developed in this study can be used to inform the development of PRO measurement strategies for clinical programs developing treatments for CLBP. Results suggest that back pain is not the only outcome that should be targeted while treating CLBP, but rather the overall daily patient experience.

## Author Contributions

**Ekin Seçinti:** conceptualization, methodology, writing – review and editing, supervision. **Brittany Klooster:** conceptualization, methodology, formal analysis, writing – original draft, writing – review and editing, visualization, supervision, funding acquisition. **Rebecca Robinson:** conceptualization, methodology, writing – review and editing. **Madison C. Bernstein:** formal analysis, investigation, writing – original draft, writing – review and editing, visualization, supervision, project administration. **Madeline Tallarico:** formal analysis, investigation, writing – original draft, writing – review and editing, visualization. **Kaitlin Chatterton:** formal analysis, writing – review and editing, visualization, supervision, project administration. **Adriana Voci:** formal analysis, investigation, writing – original draft, writing – review and editing. **Daniella Olonilua:** formal analysis, writing – review and editing. **Samantha R. Linton:** formal analysis, investigation, writing – review and editing. **Leighann Litcher‐Kelly:** conceptualization, methodology, formal analysis, writing – original draft, writing – review and editing, visualization, supervision, funding acquisition. **Marcia M. Mellado Lagarde:** methodology, writing – review and editing. **Linda A. Wietecha:** writing – review and editing. **John Markman:** writing – review and editing, supervision.

## Funding

Eli Lilly and Company provided funding for the design of this study, collection, analysis, and interpretation of data.

## Conflicts of Interest

Ekin Seçinti, Rebecca Robinson, Linda A. Wietecha, Marcia M. Mellado Lagarde and John Markman are current employees and shareholders of Eli Lilly and Company. Brittany Klooster, Madison C. Bernstein, Daniella Olonilua, and Leighann Litcher‐Kelly are employees of Adelphi Values LLC who received financial support from Eli Lilly and Company to conduct the literature review and patient interviews; Madeline Tallarico, Kaitlin Chatterton, Adriana Voci, and Samantha R. Linton were employees of Adelphi Values at the time of the study.

## Supporting information


Supporting Information S1

## Data Availability

The authors confirm that relevant data supporting the findings of this study are available within the article [and/or its supplementary materials]. Additional data that support the findings of this study are available on reasonable request from the corresponding authors.
