# Supplementary material for: Patient Experiences of Chronic Low Back Pain: Development of a Conceptual Model Based on a Targeted Literature Review and Qualitative Patient Interviews
Source: Musculoskeletal Care. 2026 Oct 28;24(4):e70281. doi: 10.1002/msc.70281 (PMC13714560; doi:10.1002/msc.70281)
Supplement: Supplementary file 1 [file MSC-24-e70281-s001.docx]

**Supplementary Materials**

| Table 1. Search strategy for MEDLINE®, Embase, and PsycINFO | | |
| --- | --- | --- |
| Step | Search terms | Hits |
| 1. | chronic low* back pain OR CLBP.kw. | 2915 |
| 2. | symptom* OR sign$1 OR impact OR function* OR health related quality life OR HRQOL OR HRQL OR QoL OR quality life OR emotion* OR (social ADJ1 activit*) OR well-being OR disability OR handicap OR mobility OR (physical ADJ1 activit*) OR (physical ADJ1 function) OR (activit* ADJ3 daily li*) OR ADL OR (work ADJ1 capacity) OR (work ADJ1 restriction).sh. | 1327279 |
| 3. | symptom* OR sign$1 OR impact*OR function*OR health related quality life OR HRQOL OR HRQL OR QoL OR quality life OR emotion* OR (social ADJ1 activit*) OR well-being OR disability OR handicap OR mobility OR (physical ADJ1 activit*) OR (physical ADJ1 function) OR (activit* ADJ3 daily li*) OR ADL OR (work ADJ1 capacity) OR (work ADJ1 restriction).ab. | 16296672 |
| 4. | 2 or 3 | 16610736 |
| 5. | 1 and 4 | 2125 |
| 6. | qualitative* OR focus group* OR interview* OR phenomenological OR grounded theory OR patient experience OR subjective experience OR patient report* OR outcome measure* OR outcome assessment* OR self-report*.sh. | 1668162 |
| 7. | qualitative* OR focus group* OR interview* OR phenomenological OR grounded theory OR patient experience OR subjective experience OR patient report* OR outcome measure* OR outcome assessment* OR self report*.ab. | 3036409 |
| 8. | 6 or 7 | 3916862 |
| 9. | 5 and 8 | 784 |
| 10. | Limit step 9 to English Language | 768 |
| 11. | Limited step 10 to human | 708 |
| 12. | Limited step 11 to adult | 521 |
| 13. | Limit step 12 to published in the last 10 years (“2013-Current”) | 392 |
| 14. | Remove duplicates from 13 | 295 |

Database searches included Embase 1996 to 2023 Week 03, Ovid MEDLINE(R) ALL 1946 to January 20, 2023, and PsycINFO 2002 to January Week 3 2023. The symptom “*” indicates unlimited truncation (i.e., performs searches for variations on a word that are formed with different suffixes). The symbol “$1” indicates limited truncation for one additional character. The characters “ADJ” indicate the use of the Defined Adjacency Operator, which retrieves documents that contain query words within a specified number (n) of words from each other in any order. The characters “.kw” indicate the search was conducted on keywords. The characters “.ab” indicates the search was conducted on abstracts. The characters “.sh” indicates the search was conducted on subject headings.

| Table 2. Eligibility criteria for the targeted literature review | | |
| --- | --- | --- |
| Study characteristic | Eligible | Ineligible |
| Patient population | - Humans with CLBP - Adults (≥18 years old) | - Humans without CLBP - Non-humans - Children and/or adolescents (i.e., age <18 years old) |
| Intervention – Treatment | - All interventions are eligible treatments | - Not applicable |
| Intervention – Comparison | - All interventions, placebo or usual care are eligible comparators | - Not applicable |
| Outcomes | - Key signs, symptoms and/or quality of life impacts of CLBP (abstracts discussing the patient perspective and/or an individual’s lived experience of these concepts will be prioritized for review) - Clinician perspective of signs, symptoms, and quality of life impacts of CLBP may also be considered | - Other aspects of chronic low back pain that do not include discussion of signs, symptoms, or quality-of-life impacts - Perspectives related to caregiver or clinician burden, or healthcare service evaluations - Pathogenesis, molecular, or biology-based findings of the disease |
| Time frame | - 2013–January 2023 | - Publications prior to 2013 |
| Study design | - Randomized controlled trials - Single group designs - Observational trials (e.g., natural history studies) - Cohort studies - Case control studies - Cross sectional designs - Data collected through qualitative methods (e.g., interview, focus group) - Literature reviews focusing on patient experience | - Case studies (1–2 patients) - Letters, editorials, commentaries, opinion pieces |
| Language | - English | - Non-English |
| Abbreviations: CLBP = chronic low back pain | | |

| Table 3. Qualitative interview study participant inclusion/exclusion criteria | |
| --- | --- |
| Inclusion criteria | Exclusion criteria |
| To be included in the study, participants must:   - Provide written informed consent for interview participation by completing a signed Consent to Release Information Form (CRIF) - Be 18 years or older - Have a physician-confirmed diagnosis of chronic low back pain - Be on a consistent treatment plan for chronic low back pain (pharmacological and/or non-pharmacological) for a month prior to the interview - Have experienced lower back pain for at least 3 months - Experience lower back pain located between the bottom of the rib cage (12^th^ thoracic vertebra) and the upper thigh (lower gluteal folder), with or without radiation into the posterior thigh (If radiation was present, must stop above the knee) - Be fluent in English (i.e., able to speak, read, write, and comprehend) - Be willing to participate in one 90-minute audio-recorded interview via telephone or teleconference | To be excluded from the study, participants have:   - A history of surgery to the lower spine (T10-S1) within 1 year before the interview (e.g., fusion, laminectomy, discectomy, foraminotomy, nucleoplasty, disc replacement, kyphoplasty) - A history of potentially causative and/or confounding sources of pain including:   - Compression fracture within 1 year before the interview   - Spinal deformity (e.g., spondylolisthesis, spondylolysis, and scoliosis)   - Extra-spinal conditions with painful radiation to the lower back (i.e., visceral, genito-urinary, vascular)   - Rheumatic disease including systemic lupus erythematosus, ankylosing spondylitis, rheumatoid arthritis, psoriatic arthritis, reactive arthritis, inflammatory bowel disease-related arthropathy   - Complex pain conditions such as fibromyalgia and chronic pain syndrome   - Neurological diseases including multiple sclerosis, Guillain-Barre, and Parkinson's disease   - Tumor or infection of spinal cord - A history of lumbosacral radiculopathy (within the past 2 years) - An experience of back pain due to recent major trauma (e.g., vertebral fracture, post-traumatic spondylolisthesis) within 6 months before the interview - A current experience in a clinical trial for treatment of CLBP - A current or pending worker’s compensation, litigation, disability, or any other monetary settlement regarding their CLBP or any other pain condition, or any closed claim within the past 5 years - An inability to largely or wholly participate fully in all protocol procedures (e.g., bedridden or confined to a wheelchair), permitting little or no selfcare - Any condition or situation that may interfere significantly with the individual’s involvement in the study in the opinion of their clinician (e.g., cognitive impairment or other medical condition) |
